# Ethylene glycol toxicosis in milk-fed dairy calves

**DOI:** 10.1186/s13028-022-00626-1

**Published:** 2022-03-24

**Authors:** Jørgen Steen Agerholm, Kirsten Søndergaard Hansen, Hanne Lerche Voogd, Anne Kirstine Havnsøe Krogh

**Affiliations:** 1 Department of Veterinary Clinical Sciences, University of Copenhagen, 1870 Frederiksberg C, Denmark; 2 Kvægdyrlægerne Midt, Klochsvej 2B, 7441 Bording, Denmark

**Keywords:** Antifreeze, Geothermal heating, Intoxication, Kidney, Milk, Milk taxi, Nephrosis, Oxalate, Poisoning, Tubular necrosis

## Abstract

**Background:**

Ethylene glycol (EG) (antifreeze) toxicosis has mostly been reported in dogs and cats, while reports on EG toxicosis in cattle are sparse. We report EG toxicosis in 25 milk-fed calves associated with a leak in the cooling pipes in a milk taxi. The milk taxi was connected to a geothermal heating system in which EG was used as antifreeze.

**Case presentation:**

Although the assistant responsible for feeding milk to the calves observed a few blue-colored droplets of liquid on the surface of the milk in the milk taxi and suspected EG contamination, the milk was fed to the calves. Within hours, the calves became depressed and some died within the next 2 days. Necropsy and histopathology revealed widespread severe acute renal tubular necrosis with numerous birefringent crystals in the tubular lumen. Biochemical analysis of serum showed severe damage to the kidneys (marked azotemia) and hypochloremia, hyponatremia and hyperkalemia; findings consisting with metabolic acidosis. After feeding the calves, the assistant inspected the milk taxi and found a leaking cooling pipe.

**Conclusions:**

The suspected EG toxicosis was confirmed by the observation of renal tubular necrosis, numerous intratubular crystals, and metabolic acidosis. EG toxicosis due to leaking pipes connected to a geothermal heating system has not been reported previously. Alternative antifreeze products that are less toxic than EG are recommended for use if there is a risk of contamination of human and animal foodstuffs in case of a leak in the system.

## Background

Ethylene glycol (EG) is widely used in the plastic and synthetic textile industry, but is generally better known for its antifreeze properties, as utilized in e.g., automotive antifreeze. Commercial antifreeze products contain around 95% EG, as well as other compounds such as anticorrosive substances and tracer color. EG has no odor or color, is water-soluble and upon ingestion, humans have reported that the taste is bitter-sweet and it leaves a slightly warm sensation in the mouth. EG itself has a toxicity equivalent to ethanol, but EG is metabolized in the liver to glycolaldehyde, glycolic acid, glyoxylic acid and oxalic acid, which have cell toxic properties. Intoxication by these metabolites causes central nervous system (CNS) depression, as well as renal and cardio-pulmonary failure [1, 2].

In veterinary medicine, EG toxicosis is most commonly reported in dogs and cats [3], although rare episodes have been reported in other domestic animal species [4–7]. EG toxicosis in cattle is rarely reported. Barigye et al. [8] reported multiple deaths after feeding EG-contaminated plant by-products to group of adult beef cattle, while Crowell et al. [9] reported an isolated case in a 1-month-old Jersey calf that had access to antifreeze. In their report, Crowell et al. mention two additional incidents of EG toxicosis in cattle; both cases also related to the consumption of antifreeze from open containers. In this case study, we report an outbreak of EG toxicosis in dairy calves fed milk contaminated by EG via a leak in the milk cooling system, and we call for awareness whenever EG is used at cattle farms.

## Case presentation

A Holstein dairy herd with 200 cattle experienced the sudden death of three out of a group of 25 milk-fed calves between May 22 and May 24, 2021. Milk used for feeding young calves in the herd was distributed by a milk taxi. The calves were fed around 3 L of whole milk twice a day. The calves had developed normally until the age of around 2 weeks, when they suddenly displayed reduced appetite and depression followed by apathy and death within one to two days. The owner regarded this as an isolated incident and did not consult the herd veterinarian or change procedures.

**Fig. 1:**
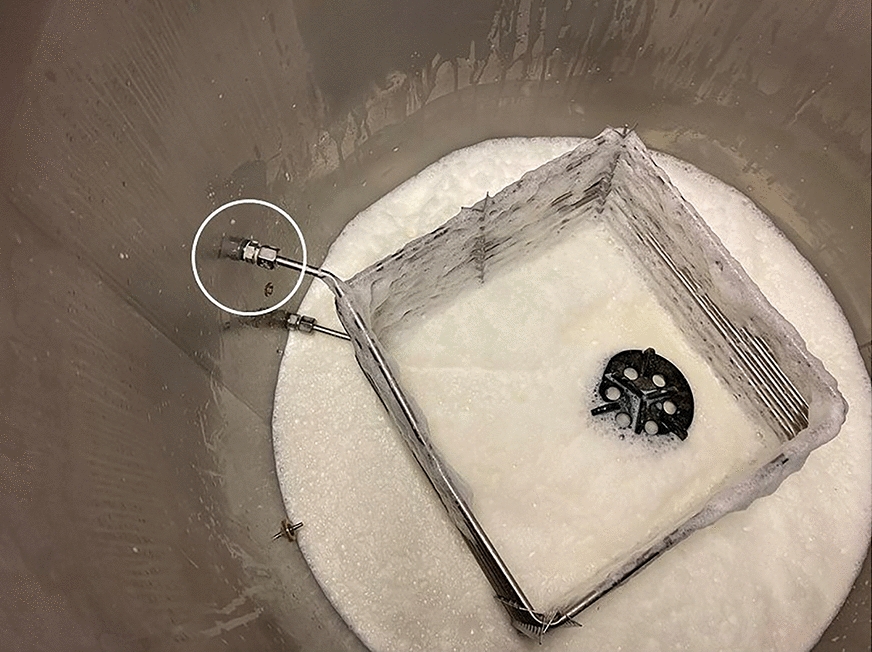
A milk taxi was used for preparing and distributing milk for the calves. The location of the leaking pipe is indicated by a circle. The leak itself cannot be seen

On June 10, the assistant responsible for feeding milk to the calves observed a small number of blue-colored droplets of liquid on the surface of the milk in the milk taxi. He suspected that the colored droplets were tracer color from the EG that was used in the herd’s bulk tank milk cooling (ice bank) system. When preparing milk for the calves, the ice bank system was temporarily connected to the milk taxi’s cooling system to ensure rapid cooling of the milk after pasteurization. The milk taxi was usually loaded with 70–80 L of milk. The assistant tasted the milk but did not detect any unexpected taste. He decided to feed the milk to the calves without knowing the toxicity of EG nor its concentration in the milk. After having fed the contents of the milk taxi to the calves, the assistant inspected the milk taxi’s cooling system and discovered a leak in a cooling pipe fitting (Fig. 1). Use of the milk taxi was immediately discontinued until the leak had been repaired. A fourth calf died on the same day with clinical signs similar to the previous three. Assistance from the herd veterinarian was requested the next day.

A fifth calf died during the night and the veterinarian found several diseased calves on June 11. The primary clinical signs were related to the CNS and consisted of stereotyped behavior, depression, paresis, seizure and salivation, with most calves only displaying stereotyped behavior and depression. The calves were not febrile. Blood samples for biochemical and hematological analyses were taken from two of the worst affected calves and submitted to LABOKLIN (Bad Kissingen, Germany). The serum biochemical analyses showed marked azotemia characterized by 4.3 and 3.7 times the upper reference limit (URL) of creatinine and 1.8 and 1.3 times the URL of urea in each calf, respectively. Electrolytes were also affected in both calves, characterized by hypochloremia (0.98; 0.97 times the lower reference limit (LRL)), hyponatremia (0.93; 0.95 times the LRL) and hyperkalemia (1.78; 1.42 times the URL). One calf also had hyperphosphatemia (1.17 times the URL). The liver-related membranous enzyme gamma glutamyltransferase (GGT) was increased in both calves (2.4 and 2.7 times the URL), while hypoproteinemia and hypoalbuminemia were also present. Hematological parameters were mildly affected and showed mild leukocytosis, neutrophilia and thrombocytosis. Complete biochemical and hematological data are shown in Tables 1 and 2.

**Table 1:** Biochemical analyses of serum from two calves with ethylene glycol toxicosis

Parameter	Calf 1	Calf 2	Reference interval^a^
AP (U/L)	157.00	**336.00**	< 300
GGT (U/L)	**47.60**	**54.00**	< 20
GLDH (U/L)	7.30	5.30	< 8
Bilirubin total (µmol/L)	3.40	3.60	< 5
Cholesterol (mmol/L)	**1.40**	**1.40**	2.07–3.88
Triglycerides (mmol/L)	0.16	0.13	0.17–0.51
AST (U/L)	17.00	25.50	< 80
LDH (U/L)	616.90	783.70	< 1500
CK (U/L)	32.00	49.00	< 250
Total protein (g/L)	**51.40**	**48.00**	60–80
Chloride (mmol/L)	**88.00**	**87.00**	90–110
Albumin (g/L)	**27.90**	**26.90**	30–40
Globulins (g/L)	23.50	21.10	< 48
Urea (mmol/L)	**14.50**	**10.70**	< 8
Creatinine (µmol/L)	**756.00**	**657.00**	88–177
Phosphate (mmol/L)	**2.80**	2.10	1.1–2.4
Calcium (mmol/L)	2.70	2.60	2.3–2.8
Magnesium (mmol/L)	1.00	0.90	0.8–1.3
Potassium (mmol/L)	**8.00**	**6.40**	3.5–4.5
Sodium (mmol/L)	**126.00**	**128.00**	135–145
Iron (µmol/L)	**11.20**	**43.80**	20–40
Zinc (µmol/L)	15.40	18.70	8–24
Copper (µmol/L)	12.50	10.50	8–24
Selenium (µg/L)	44.10	55.20	40–85

*AP* alkaline phosphatase, *GGT* gamma-glutamyl transferase, *GLDH* Glutamate dehydrogenase, *AST* Aspartate aminotransferase, *LDH* Lactate dehydrogenase, *CK* Creatine kinase

Values outside the reference interval are indicated in bold

^a^According to LABOKLIN, Bad Kissingen, Germany

**Table 2:** Hematological analyses of two calves with ethylene glycol toxicosis

Parameter	Calf 1	Calf 2	Reference interval^a^
Blood count^b^			
Erythrocytes (T/L)	8.83	7.12	5.0–10.0
Hematocrit (L/L)	0.35	0.32	0.28–0.38
Hemoglobin (g/L)	116.00	97.00	90–140
Leukocytes (G/L)	**11.30**	8.90	4.0–10.0
Neutrophils (%)	49.00	60.00	25–45
Lymphocytes (%)	48.00	37.00	45–65
Monocytes (%)	1.00	1.00	2–6
Eosinophils (%)	1.00	1.00	1–10
Basophiles (%)	1.00	1.00	0–2
Band neutrophils (%)	0.00	0.00	0–3
Hypochromasia (yes/no)	**Yes**	No	No
Anisocytosis (yes/no)	No	No	No
Thrombocytes (G/L)	**907.00**	643.00	300–800
Differential blood count (absolute)			
Neutrophils (G/L)	**5.50**	**5.30**	1.0–3.5
Lymphocytes (G/L)	5.40	3.30	2.5–5.5
Monocytes (G/L)	0.10	0.10	0.0–0.33
Eosinophils (G/L)	0.10	0.10	0.3–1.5
Basophils (G/L)	0.10	0.10	0.0–0.1
Bands 0.00 (G/L)	0.00	0.00	0.0–0.2

Values outside the reference interval are indicated in bold

^a^According to LABOKLIN, Bad Kissingen, Germany

^b^Laser light scattering method/microscopical

**Fig. 2:**
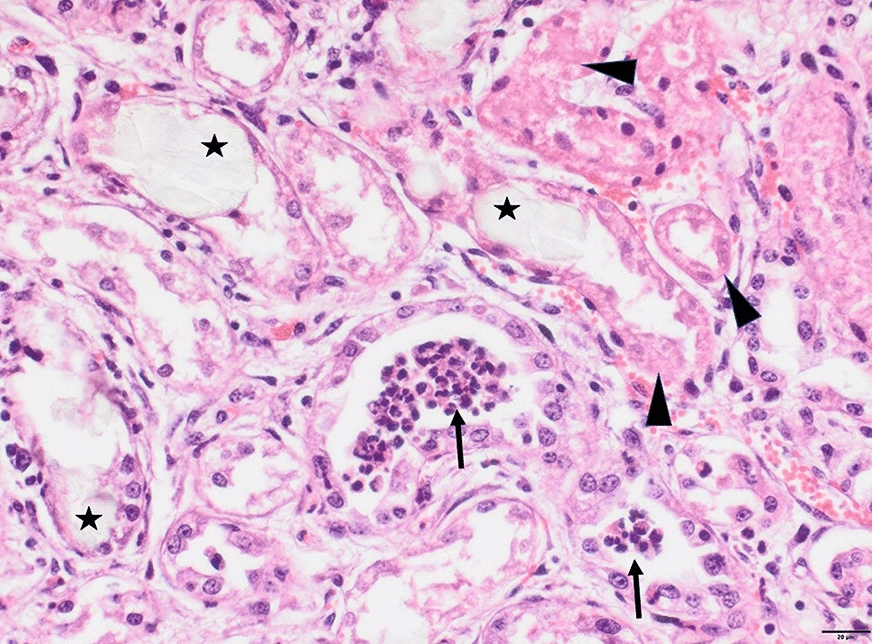
Photomicrograph showing acute renal tubular necrosis (*arrowhead*) and intratubular crystals (*asterisk*) associated with a loss of tubular epithelium. Necrotic cells (probably representing necrotic tubular epithelial cells) are present within a tubular lumen (*arrow*). Calf, hematoxylin and eosin. Bar: 20 µm

**Fig. 3:**
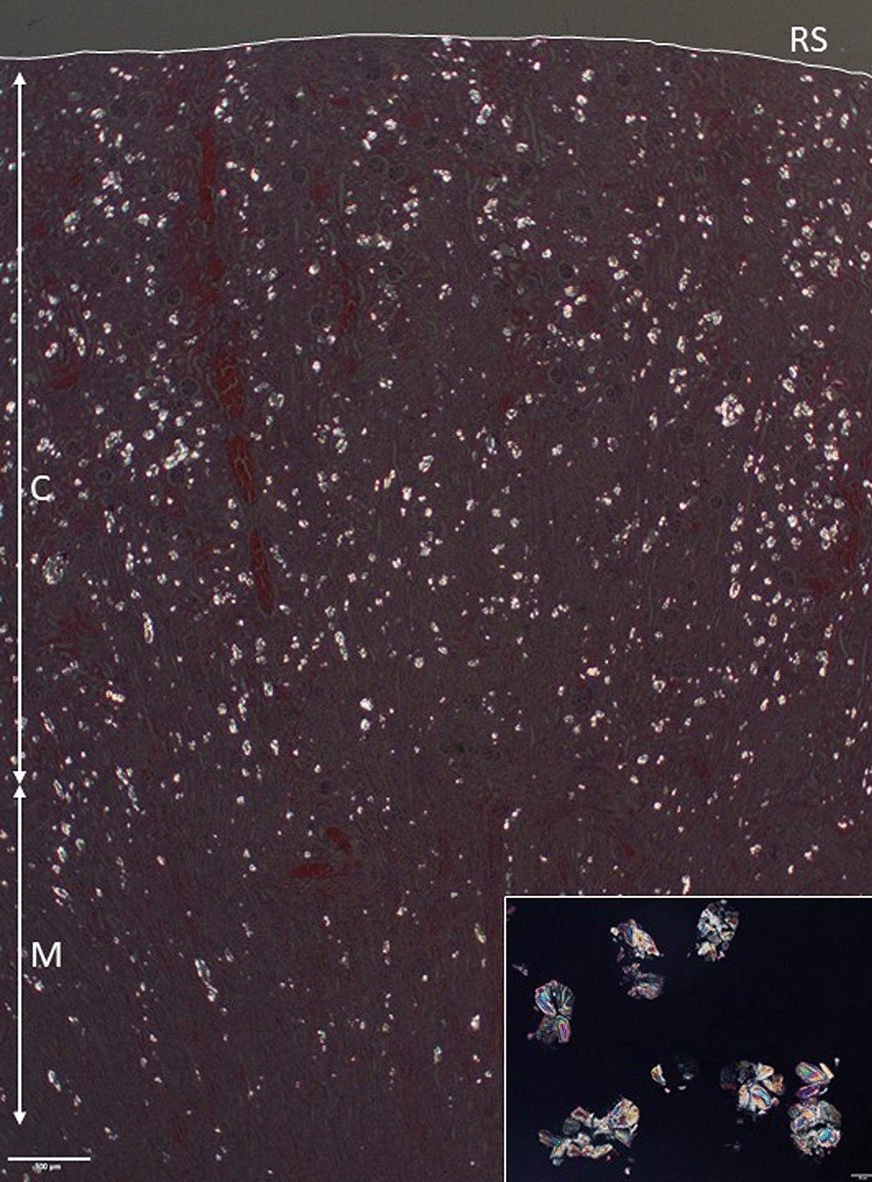
Photomicrograph of a kidney section seen after polarization of the light. Numerous birefringent crystals are visible, especially in the cortex. Insert: Higher magnification showing oxalate crystals. RS: renal serosa; C: cortex; M: medulla. Bar: 500 µm

Two of the recently dead calves were necropsied at the farm. The kidneys were enlarged and edematous with a soft cortex, while the liver was slightly enlarged. No other lesions were observed in the thoracic and abdominal organs. Specimens of the kidneys and liver were fixed in 10% neutral buffered formalin for histology and a sample of urine was taken for microscopy. Microscopy of urinary sediment after centrifugation revealed the presence of numerous calcium oxalate crystals. Histology of hematoxylin and eosin stained 4–5 µm paraffin embedded tissue sections of the kidneys showed severe widespread acute tubular necrosis, dilated tubuli and sloughing of necrotic tubular epithelial cells into the tubular lumen. Intratubular pale yellow crystals were widespread and associated with necrosis and loss of the tubular epithelium (Fig. 2). Viewing under polarized light revealed numerous birefringent crystals arranged in sheaves or rosettes. The crystals were more numerous in the renal cortex than in the medulla (Fig. 3). One of the calves also had renal interstitial fibrosis and lymphocytic infiltrations. The liver of one of the calves had scattered single-cell necrosis, while the other had mild steatosis.

Apart from avoiding any additional ingestion of EG, no specific treatment was initiated in the herd. Over the following days (until June 15), an additional 6 calves died, giving a total loss of 11 calves out of the 25 calves exposed to EG. The remaining calves recovered without displaying long-term clinical complications.

## Discussion and conclusions

In the present case, a presumptive diagnosis of EG toxicosis was made based on the observation of blue-colored droplets in the milk fed to calves that subsequently became sick. A blue-colored EG-based antifreeze had been added to the milk cooling system. The diagnosis was confirmed by microscopic observation of renal tubular necrosis and the presence of numerous intratubular crystals; findings that are virtually pathognomonic for EG toxicosis [10]. The presence of metabolic acidosis indicated by hyperkalemia, hyponatremia, and hypochloremia, and renal damage reflected by azotemia combined with hyperphosphatemia in one calf supported this diagnosis.

The observed kidney-related biochemical changes can be caused by a decreased glomerular filtration rate, tubular destruction and decreased resorption of analytes [11]. In addition, increased GGT activity was identified, a finding that may be caused by toxic EG metabolites affecting the liver [12, 13]. However, GGT can also be released from proximal renal tubular epithelial cells but would then be expected to enter the urine rather than passing into the blood [14]. The total protein and albumin concentrations were decreased, while the globulin concentration was within reference interval and similar to levels found in healthy Holstein calves evaluated for age-related changes in biochemistry values [15]. The decreased protein and albumin concentrations may be explained by a combination of decreased production by the liver and increased loss of plasma albumin through the kidneys.

In contrast to the biochemical changes, the hematologic findings were mild with signs of stress-related changes, leukocytosis and neutrophilia, which have previously been identified in dogs and cats with EG intoxication [11, 16].

The susceptibility of cattle to EG intoxication is dependent on their age, as susceptibility decreases with increasing efficiency of ruminal fermentation. In an experimental study, a single oral dose of 2 mL to 5 mL of EG/kg body weight (BW) given to calves weighing 26–34 kg caused their deaths within three to six days. However, the toxicity of a single lower dose was not investigated, nor was the impact of repeated exposure to lower doses [9]. It is therefore possible that much lower doses than 2 mL/kg BW or repeated exposure to lower doses would be toxic or even lethal to non-ruminating calves. The exposure dose in the present study could not be determined as the amount that leaked into the milk was unknown. However, it is likely that the calves were exposed repeatedly between May 22 and June 10, at least until a calf became clinically affected and stopped drinking milk. However, the exact exposure of each calf remains unknown.

EG is rapidly absorbed across the gastro-intestinal mucosa in mono-gastric species—and presumably also in non-fermentating ruminants—and plasma concentration peaks within a few hours. EG is not particularly toxic by itself and a significant amount is excreted unchanged in the urine within a few hours. However, EG is metabolized in the liver through a number of steps: it is first metabolized to glycoaldehyde, which is rapidly converted to glycolic acid. Glycolic acid is responsible for the severe metabolic acidosis that develops in cases of EG toxicosis [11], which was also observed in our case. Treatment of metabolic acidosis is essential for survival in the acute stage. Glycolic acid is metabolized to glyoxylic acid, which is toxic but has such a short half-life that toxic levels will not be reached. Glyoxylic acid can be metabolized through several pathways, with the formation of oxalic acid being of major significance. Oxalic acid is excreted through the urine, but precipitates with calcium in the glomerular ultrafiltrate to form calcium oxalate when pH decreases. Calcium oxalate crystals may damage the tubular epithelium and cause tubular obstruction, which is of particular importance for animals surviving acute toxicosis. However, precipitation may also lead to other pathological conditions such as low plasma calcium level [17]. In dogs and cats, hypocalcemia can be seen in approximately half of the cases [11]. The two calves investigated in our study had calcium concentrations within the reference intervals (Table 1).

Renal tubular damage in EG toxicosis is the result of two processes: toxic metabolites cause degeneration and necrosis of the tubular epithelium, while calcium oxalate crystals in the tubular lumen lead to the degeneration of epithelial cells and—depending on their numbers and size—may cause obstruction of the urinary flow. However, as the tubular basement membranes remain intact, regeneration can occur if the animal survives the acute toxicosis. If exposure to the nephrotoxin is discontinued, a normal-appearing tubular epithelium will return in seven to 14 days after last exposure, and the renal morphology will be completely normal 21 to 56 days after last exposure [18]. In accordance with this, the calves that survived the acute toxicosis recovered.

The milk taxi’s cooling system was constructed in such a way that it could be connected to the ice-bank system of the farm’s bulk milk cooling tank to reduce water consumption and ensure rapid cooling of the milk after pasteurization. The cooling system of the ice-bank was connected to a geothermal heating system on the property, which contained several hundred liters of water with an EG concentration of around 30%. Adding antifreeze to the geothermal heating system is a requirement from the manufacturer. Due to financial reasons, EG was used as antifreeze at this property, instead of the more expensive propylene glycol or other less toxic compounds. This case illustrates the hazard associated with using EG in systems where the substance can come into direct contact with animal or human foodstuffs in case of leaking pipes, etc., and calls for awareness among veterinarians, agricultural advisors, technicians and farmers.

## Data Availability

The datasets used and/or analysed during the current study are available from the corresponding author on reasonable request.
